# The effects of a group-based intervention through physical activities and dietary changes in young patients with severe psychiatric disorders: a pilot study

**DOI:** 10.3389/fspor.2023.1197925

**Published:** 2023-08-22

**Authors:** Othman Sentissi, Gabrielle Zosso, Anouck Cogordon, Chiara Chillà

**Affiliations:** ^1^ Adult Psychiatric Division, Ambulatory Psychiatric Centre (Cappi Jonction), University Hospitals of Geneva, Geneva, Switzerland; ^2^ Department of Psychiatry, Faculty of Medicine, University of Geneva, Geneva, Switzerland

**Keywords:** physical activity program, exercises, dietary program, eating behaviour, mental disorders, weight gain, metabolic disturbances

## Abstract

**Background and objectives:**

The present study aims to investigate the effect of the 4-F (Fit, Fun, Feel, and Food) group-based program on physical, clinical, and biological outcomes in young patients suffering from severe psychiatric disorders.

**Methods:**

A pilot study with a naturalistic design was conducted to investigate the effect of a group-based intervention on young patients.

**Results:**

A descriptive analysis revealed that out of the 61 outpatients initially enrolled in the program, with a mean age of 26.9 years old (±6.1, 60% men), 71% were overweight or obese. Paired T-tests for the difference between T0 and T1 were used to evaluate the evolution of the outcomes. The 24 patients who completed the full program showed no significant decrease in weight or body composition. Despite the limitations, the main findings of this study were the significant improvement in muscular endurance and coordination (from T0 (*M* = 13.65, SD = ±1.93) compared to T1 (*M* = 12.49, SD = ±1.81), [*t*(20) = 3.072, *p* < 0.05] and the general increase in mental well-being from baseline to the end of the program according to the type of psychopathology [*F*(3,10) = 4.25, *p* < .05]. A slight modification in eating behavior, with a tendency towards a decrease in TFEQ hunger levels, was also noticed. The ANCOVA showed no difference in outcomes between the groups based on diagnosis.

**Conclusion:**

Despite its limitations and the small sample size, this pilot study provides valuable insights, demonstrating the feasibility of the program and its positive impact on physical well-being and improved mental health in young patients with psychiatric disorders, sedentary behavior, and unhealthy lifestyles. These encouraging results warrant further research in controlled, larger population samples to deepen our understanding of the potential effects of such interventions.

## Introduction

People with moderate to severe mental illness have higher rates of somatic comorbidities and premature mortality than the general population (1, 2). Factors such as individual vulnerability, an unhealthy lifestyle, a poor diet, a lack of exercise, and sedentary behavior (3, 4), in addition to the use of new atypical antipsychotics as first-line therapy (5, 6), may lead to the rapid onset of weight gain (7). Recent studies in this field have reported that weight gain is only the beginning of a series of comorbidities such as metabolic disorders, metabolic syndrome (MetS), and premature cardiovascular complications (8, 9). The short-term consequence is poor adherence to treatment (10), while the medium-to-long-term consequences include physical or mental discomfort, impaired quality of life, and decreased life expectancy compared to the general population (11).

New evidence suggests that balanced dietary habits, physical activity (PA), and other lifestyle modifications may modulate brain function and promote changes in adaptive neural plasticity (12). In an incidence study (NEMESIS), it was observed that patients with mental illness who engaged in regular PA were more likely to recover from their illness compared to their counterparts who did not exercise (13), and the patients had enhanced mental capacity and cognitive function (14, 15). There is biological plausibility that exercise may have anti-inflammatory effects (16), and that PA allows for improvements in functional ability, cardiorespiratory fitness, and overall physical health (17). Lifestyle and behavioral interventions were demonstrated to be effective in reducing weight after 18 months in a large-scale study of obese individuals with severe mental illness (18). Weight loss can be achieved by combining an appropriate exercise program with healthy eating behaviors rather than by dietary restriction alone (19, 20). The involvement of a qualified professional in exercise interventions for a population of 594 patients with schizophrenia, with a mean age of 37.2 years, was observed to be significantly associated with lower dropout rates, higher-intensity physical activity, and improved adherence to treatment and group cohesion (21).

Psychiatric disorders usually begin during adolescence and early adulthood (6). The neurobiological etiology of the positive impact of PA on the psychiatric symptoms of schizophrenia remains unclear (22). However, the prevalence of excess body fat may be up to four times higher in this population than in the general population (mean age: 41 years) (23). This is especially true in patients with first-episode psychosis with a mean age of 22.2 years, as reported in an early intervention study, in whom providing information about diet and exercise may not be sufficient to prevent weight gain; thus, the need to implement effective lifestyle and life skills interventions as routine care from the beginning of treatment in young people with severe mental illness is crucial (24). Similarly, a randomized control study observed better weight management in the intervention group (behavioral interventions, diet, and exercise) within the first 3 months following the introduction of atypical antipsychotics in drug-naïve first-episode psychotic patients. These results were confirmed in a 14-week aerobic interval training program in 25 male subjects, aged 18–35 years, following their first episode of psychosis. The authors observed the effectiveness of aerobic training in reducing weight and waist circumference while improving maximal oxygen uptake (38% increase in VO_2_ max and a 9 bpm decrease in resting heart rate) (25).

Furthermore, several clinical studies have investigated PA programs and suggested that exercise can relieve both depressive and anxiety disorders. An association between depression, sedentary behavior, and lack of PA has previously been reported (26). Based on 23 randomized controlled trials comparing physical exercise with different conditions (usual care, waiting lists, or no intervention), this meta-analysis showed that exercise had a moderate to large and significant effect on depressive symptoms in adult participants aged 18 and older compared with usual care and the control group, but the effect was not significant compared with psychological treatment and medication (27). A 12-week moderate-intensity exercise intervention showed a positive effect, with a reduction in symptoms and severity of anxiety and an increase in overall well-being. Cardiorespiratory fitness improved over a relatively short period in 15 patients suffering from obsessive-compulsive disorder (28). These results were in line with a randomized controlled trial that reported that anxiety decreased in the intervention group benefiting from PA and a sedentary lifestyle increased the risk of developing anxiety with cognitive and psychosocial changes in 39 young adults with anxiety aged 18–35 years in a 2-week program (1 week sedentary and 1 week PA with a pedometer) (29).

Finally, a group-based program should improve medium-to-long-term adherence to healthy habits, resulting in better compliance with appropriate psychotropic treatment, better quality of life, prevention of weight gain, and metabolic and cardiovascular outcomes (30).

It is challenging in clinical psychiatric practice to focus on improved physical and mental health conditions; however, we believe that it is crucial to investigate the effects of a multidisciplinary, group-based program aimed at improving the physical, nutritional, and mental well-being of young patients suffering from psychiatric disorders. Based on the current knowledge of the positive effects of regular PA on anxiety, depression, and psychotic symptoms, the 4-F program was established in the psychiatric department (PD) of the University Hospitals of Geneva, focusing on physical activity, education on healthy eating, and improved body perception and emotion regulation (20, 31).

In addition, we recently conducted a scientometric analysis of PA, mental health, and well-being to evaluate key themes and trends over the past decades. Our results strengthen and expand the central role of physical activity in public health and call for an optimization of the implementation of physical activity in health policies (32).

We can therefore assume that such an intervention can prevent weight gain and improve mental and physical health in young patients aged 18–35 years with severe psychiatric disorders. On this basis, we conducted a pilot study with a naturalistic design to investigate the effect of a group-based intervention.

## Materials and methods

### Aim of the study

The aim of this pilot study was to investigate the feasibility of the 4-F program (Fit, Fun, Feel, and Food) and to assess the effect of this group-based intervention on physical, clinical, and biological outcomes over time in a cohort of 24 young outpatients with moderate-to-severe psychiatric disorders.

The 4-F program was set up in June 2017 in the psychiatric department (PD) of the University Hospitals of Geneva to optimize the measures and strategies needed to prevent and avoid weight gain and metabolic disorders in young patients with psychiatric disorders. This departmental program is naturalistically and systematically focused on PA, a healthy diet, and improved body perception.

**Figure 1:**
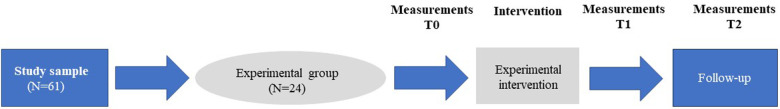
Experimental design of the study.

This pilot study was conducted over three evaluation periods of the program: Visit 1 at the beginning and Visit 2 at the end of the program (at baseline and after 8 weeks), and one follow-up (phone call) 12 weeks after the inclusion date (Figure 1). Clinical and anthropometric evaluations were part of routine practice; blood tests and an ECG were performed under the usual conditions.

### Participant selection

From August 2018 to October 2020, we recruited outpatients in an ambulatory mental health center in Geneva, patients with moderate-to-severe psychiatric disorders [MINI International Neuropsychiatric Interview (33)], both men and women, aged between 18 and 35 years, treated or untreated with psychotropic medication. Almost all patients included in the study also had a predisposition to weight gain (BMI, Metab Sd), were medically able to participate in an exercise program, and agreed to sign an informed consent form. We included patients with severe psychiatric mental disorders meeting the DSM-5 criteria (MINI International Neuropsychiatric Interview; Sheehan et al., 1998) with the medical ability to participate in an exercise program without decompensated somatic disorders. Exclusion criteria for the study comprised individuals with severe personality or addictive disorders, individuals with acute somatic disorders, or severe suicidal risk, and those who did not speak French.

### Data collection and procedures

The monitoring was centered and conducted in a naturalistic and systematic way by qualified professionals consisting of senior psychiatrists, psychologists, PA coaches (qualified personnel who followed the exercise program), dieticians, and registered nurses. This collaboration involved the physicians of these patients.

### Outcome parameters

A special study procedure was set up in addition to the usual 4-F program procedure (31). In addition to the interview and series of evaluations used in routine program inclusion practice, and psychometric measures evaluations (such as the MINI International Neuropsychiatric Interview and follow-up telephone interview, MADRS (34), HAMA (35) psychotic disorders (PANSS; respectively, Kay et al., 1987 (36), well-being [WEMWBS (37)]. Evaluation of eating behaviors of [TFEQ (38) were carried out]. Blood samples (fasting blood glucose and lipid levels) were measured in the morning at baseline and T1 using standard laboratory procedures.

The vital signs, height, weight (using a balance-beam scale), BMI (kg/m^2^), umbilical perimeter, VO2 max (an index of an individual's maximum capacity to transport and use oxygen during incremental aerobic exercise), and body composition (biceps, triceps, subscapularis, supra-iliac) were recorded using a Body stat impedance meter at baseline and T1 with the same conditions prior to testing*.* An ECG was obtained for all patients in the study upon inclusion.

To assess the patients' activity and maintenance of lifestyle habits, we performed a follow-up telephone interview 1 month after the end of the study (T2) to evaluate the patient's physical activity, body weight, and clinical condition [CGI (39)].

### Interventions and procedures

As part of the 4-F program, patients participated in two sessions of moderate exercise intervention for 60 min each per week. One of the sessions (“Fit”) consisted of a progressive warm-up and moderate-intensity aerobic exercise in the form of circuit training. The other session (“Fun”) consisted of a warm-up followed by a chosen sport (basketball, handball, soccer, etc.) at moderate-to-sustained intensity (the intensity of the physical activity was assessed by the coach based on their experience without the use of specific equipment). Patients were assessed with the Eurofit physical fitness test battery (European Physical Fitness Tests, EUROFIT Experts' Committee on Sports Research, 1993) (40) to evaluate cardiorespiratory endurance, coordination, suppleness, and balance (e.g., shuttle run test for cardiorespiratory endurance or plate strike test for limb speed).

A therapeutic session (“Feel”) with interdisciplinary co-facilitators (a psychologist and a dietician) provided a therapeutic space for talking: participants shared their experiences with weight gain and their body perception and exchanged information about their therapeutic program. The facilitators had the dual objective of motivational interviewing and nutritional listening on the one hand and psychological support (through self-esteem and support, for example) on the other.

The dietician and the psychologist organized bimonthly sessions (“Food”) to share educational counseling on nutrition knowledge and behavioral goals according to well-defined topics. The bimonthly sessions helped the participants share their experiences with eating habits, weight gain, and healthy lifestyles.

#### Data analysis

Descriptive analysis of the data was expressed as mean (±SD), percentage, or number of patients. Paired T-tests for the difference between T0 and T1 (8 weeks after the beginning of the program) were used to evaluate the evolution of anthropometric (fat mass per kg, fat-free mass per kg, abdominal circumference, weight, BMI, waist circumference, blood pressure, waist-to-hip ratio, and hip circumference), psychometric (WEMWBS, MADRS, PANSS), and nutritional (TEFQ) outcomes.

In addition, a general linear model of covariance (ANCOVA) was first performed to examine between-group differences in anthropometric, psychometric, and nutritional outcomes in the three groups of patients (patients with anxiety and depression disorders, psychotic disorders, and others). The ANCOVA allowed for controlling for gender, and a pairwise comparison with a Bonferroni-adjusted *post hoc* test was performed as a follow-up to examine the between-group effect.

Global statistical significance was set at *p* ≤ 0.05. Statistical and demographic analyses were performed with the Statistical Package for Social Sciences (SPSS) software version 25.0. (IBM Corp., Armonk, NY, USA).

### Protection of human participants

The study was conducted in accordance with the protocol and the principles of the current version of the Declaration of Helsinki and the guidelines approved by the local ethics committee of Geneva (CCER, N° 2018-00010).

## Results

### Descriptive analyses

From August 2018 to October 2020, 61 patients with a mean age of 26.9 years old (±6.1, 60% men) were included in the 4-F program. Among these patients, 73% were overweight or obese (26% and 47%, respectively), with a MetS prevalence of 39.3%, and 57.4% suffered from anxiety and mood disorders, while 26.2% suffered from psychotic disorders. Most of the patients received at least one psychotropic treatment (88.6%; and 44.3% were taking an antipsychotic) (Table 1).

**Table 1:** Description of participants at baseline (*N* = 61).

Demographic and clinical variables	Men (*N* = 37)	Women (*N* = 24)	Total (*N* = 61)
Age (year, M, SD)	26.72 (±5.99)	27.29 (±6.43)	26.95 (±6.1)
VO2 Max (M, SD)	31.38 (±−8.11)	29.78 (±2.72)	29.98 (±8.39)
Metabolic outcomes			
BMI (M, SD)	29.42 (±−6.50)	27.07 (±−6.12)	28.49 (±−6.40)
≤24.9 kg/m^2^% (*N*)	18.9 (7)	45.5 (11)	26 (16)
25–30 kg/m^2^% (*N*)	40.5 (18)	20.9 (5)	26 (16)
≥30 kg/m^2^% (*N*)	40.6 (15)	33.6 (8)	47 (29)
MetS % (*N*)	54.16 (13)	45.83 (11)	39.34 (24)
AC (cm, M, SD)	102.35 (±17.61)	91.50 (±17.30)	98.08 (±18.15)
BP (mm/Hg, M, SD)	136.2 (±13.0)	116.30 (±1.61)	128.40 (±17.2)
	85.13 (±10.4)	75.83 (±1.17)	81.48 (±11.8)
TG (mmol/l, M, SD)	2.42 (±2.72)	1.38 (±0.64)	1.98 (±2.17)
HDL-Cs (mmol/l, M, SD)	4.83 (±1.18)	4.62 (±0.98)	4.74 (±1.09)
FBS (mmol/l, M, SD)	4.89 (±0.65)	5.12 (±1.35)	4.98 (±0.98)
Clinical outcomes and treatment			
Main psychiatric diagnosis % (*N*)			
Anx, MDs	56.7 (21)	58.3 (14)	57.4 (35)
PSD, SCZ	32.4 (12)	16.7 (4)	26.2 (16)
Other	10.9 (4)	25 (6)	16.4 (10)
CGI_Gravity (M, SD)	4.33 (±1.28)	4.00 (±1.05)	4.24 (±1.03)
CGI_Improvement (M, SD)	2.78 (±1.11)	3.00 (±1.73)	2.88 (±1.46)
Treatment % (*N*)			
Antipsychotic	51.4 (19)	33.3 (8)	44.3 (27)
Other	32.4 (12)	62.5 (15)	44.3 (27)
No treatment	16.2 (6)	4.2 (1)	11.5 (7)

Data are expressed as mean (M), standard deviation (±SD), percentage (%), and number of patients (*N*).

MetS, Metabolic Syndrome, (at least three of the following five criteria: abdominal circumference, blood pressure, triglycerides, HDL cholesterol, fasting blood sugar); MetS based on the International Diabetes Federation (IDF); AC, Abdominal Circumference; BP, Blood Pressure; TG, Triglycerides; TOT-Cs, Total Cholesterol; Anx, Anxiety; MDs, Mood Disorders; PSD, Psychotic Spectrum Disorder; SCZ, Schizophrenia; HDL-Cs, High-density lipoprotein cholesterol; CGI, Clinical Global Impression.

Due to the COVID-19 outbreak, the lockdown periods, and the usual dropout rate (20%–50% in outpatients) (21, 41), almost 50% of the patients failed to complete the study protocol, and 24 patients dutifully followed the 8-week training program. We decided to investigate the feasibility of the 4-F program with a pilot study to analyze the effect of the program over time only on this sample of the population.

Of the 61 outpatients enrolled, 24 completed the 4-F program (mean participation = 33.3%), and 11 completed at least 50% of the training sessions (mean participation = 57.4%). Reasons for dropping out were lack of motivation and attrition (*N* = 8), the COVID-19 outbreak and national quarantine period (*N* = 8), worsening of psychiatric status and hospitalization (*N* = 8), and a few patients declining to start the program (*N* = 6). For the patients enrolled before the COVID-19 outbreak (January 2020) and the consequent lockdown measures, we decided to adopt the 4-F program via online videoconference format (42). Moreover, we only included the program patients from the ambulatory mental health centers. There was only one patient who was hospitalized in the last month before inclusion, and we did not find any effect of the hospitalization on clinical or physical outcomes.

The sociodemographic and clinical characteristics of this cohort of patients (*N* = 24) are shown in Tables 2, 3.

**Table 2:** Description of participants at baseline (*N* = 24).

Demographic and clinical variables	
Age (year, M, SD)	25.76 (±7.5)
Gender *(*Male) % (*N*)	66.7 (16)
Marital status (single) %	93.8
Nationality %	
CH	31.3
EU	25
AFR	12.5
AMS	18.8
Other	12.5
Educational background %	
Mandatory school	12.5
Apprenticeship	25
College	25
Graduate studies	31.3
Other	6.3
Professional activity %	
Employed/In school	40
Unemployed	13.3
Other	46.7
Social status (disability insurance/social assistance)	71.4
Living %	
Alone	25
With parents/family	43.8
Other	31.3
Cigarette/day %	
0	62.5
1–8	18.8
≥8	18.9
Coffee/day %	
0	43.8
1–3	43.8
4–5	12.5
BMI % (*N*)	
≤24.9 kg/m^2^	20.8 (5)
25–30 kg/m^2^	25 (6)
≥30 kg/m^2^	54.2 (13)
MetS (Male) % (*N*)	37.5 (16)
Psychiatric diagnosis %	
Anx, MDs	62.5
SZ, PSD	33.3
Other	4.2
Treatment % (*N*)	
Antipsychotic	54.2 (13)
Other	33.3 (8)
No treatment	12.5 (3)

Data are expressed as mean (M), standard deviation (±SD), percentage (%), and number of patients (*N*).

MetS, Metabolic Syndrome, (at least three of the following five criteria: abdominal circumference, blood pressure, triglycerides, HDL cholesterol, fasting blood sugar); Anx, Anxiety; MDs, Mood Disorders; PSD, Psychotic Spectrum Disorder; SZ, Schizophrenia; HDL-Cs, M, men; CH, Switzerland; EU, Europe; AFR, Africa.

**Table 3:** Outcomes over time (T0–T1).

Anthropometric and clinical outcomes	Pre-intervention T0	Post-intervention T1 (8 weeks after)	95% CI	*P*-value
Anthropometric outcomes (M, SD)				
Weight (kg)	88.40 (±21.08)	88.5 (±22.02)	[−3.69, 0.73]	0.18
BMI (kg/m^2^)	M 31.15 (±6.47)	M 31.90 (±7.17)	[−1.92, 0.41]	0.19
	W 29.20 (±7.53)	W 28.91 (±7.84)	[−0.69, 0.79]	0.87
	T 30.50 (±6.74)	T 31.09 (±7.30)	[−1.38, 0.31]	0.20
Fat mass (kg)	M 25.30 (±3.21)	M 25.25 (±12.11)	[−3.95, 0.38]	0.10
	W 27.71 (±4.53)	W 25.28 (±12.07)	[−0.86, 2.26]	0.30
	T 26.51 (±2.78)	T 25.26 (±11.76)	[−2.63, 0.55]	0.19
Fat-free mass (kg)	M 71.29 (±9.5)	M 70.31 (±8.99)	[−1.82, 1.56]	0.10
	W 47.33 (±5.93)	W 46.22 (±5.45)	[−1.19, 0.17]	0.12
	T 63.30 (±14.25)	T 63.09 (±13.83)	[−1.82, 1.56]	0.66
WC (cm)	M 97.06 (±14.4)	M 98.13 (±13.78)	[−3.41, 1.28]	0.35
	W 83.38 (±14.16)	W 82.67 (±13.85)	[−2.40, 1.73]	0.70
	T 92.50 (±15.48)	T 93.90 (±15.19)	[−2.57, 0.85]	0.18
AC (umbilical perimeter)	M 107 (±4.36)	M 107.19 (±18.63)	[−3.47, 3.09]	0.91
	W 96.63 (±6.16)	W 94.99 (±17.27)	[−4.63, 5.96]	0.76
According to diagnosis:	T 101.93 (±17.48)	T 103.68 (±18.80)	[−2.51, 2.60]	0.97
Anx-MD	96.60 (±22.21)	95.80 (±22.72)	[−1.42, 3.02]	0.37
Psychosis	101.70 (±18.43)	105.38 (±20.71)	[−0.27, 4.77]	**0.07**
BP (mm/hg)	133.90 (±1.47)/86.80 (±0.98)	133.60(±1.67)/85.30 (±1.04)	[−0.70, 0.65]	0.57
Waist-to-hip ratio	M 0.87 (±0.069)	M 0.87 (±0.018)	[−0.01, 0.03]	0.18
	F 0.80 (±0.085)	W 0.78 (±0.030)	[−0.01, 0.03]	0.15
	T 0.85 (±0.080)	T 0.82 (±0.018)	[−0.01, 0.03]	0.11
Hip circumference (cm)	M 110.69 (±12.27)	M 112.13 (±12.86)	[−5.04, 2.16]	0.41
	W 103.88 (±15.72)	W 104.66 (±14.88)	[−10.38, 3.71]	0.28
	T 108.42 (±13.57)	T 110.09 (±13.48)	[−4.90, 0.99]	0.57
Psychometric outcomes (M, SD)				
WEMWBS Scale—Psychopath:	44.23 (±7.8)	47.41 (±6.7)	[−8.08, 2.94]	**<0.05**
Anx-MDs	47.75 (±5.12)	51.00 (±4.94)	[−7.53, −0.97]	**<0.05**
Psychosis	41.50 (±8.96)	47.00 (±9.44)	[−13.56, 26.06]	**<0.05**
MADRS scale (only for Anx-MD patients) (M, SD)	1.75 (±0.89)	1.30 (±0.81)	[−2.25, 2.65]	0.81
PANSS scale (only for psychotic patients)				
TOT (M,SD)	1.95 (±0.39)	3.08 (±1.74)	[−3.1, 2.5]	0.21
Nutritional outcomes (M, SD)				
TFEQ				
Disinhibition	7.31 (±3.5)	6.38 (±3.5)	[−0.94, 1.55]	0.18
Hunger	7.38 (±3.9)	5.3 (±3.2)	[−0.11, 3.80]	**0.06**
Dietary restriction	8.88 (±5.0)	8.94 (±4.1)	[−1.67, 2.29]	0.95
Physical outcomes (M, SD)				
Eurofit				
Whole body balance with the “flamingo balance” test	17.95 (±14.27)	16.43 (±14.59)	[−7.40, 8.02]	0.93
Speed of limb movement by “plate tapping”	13.65 (±1.93)	12.49 (±1.81)	[0.09, 2.13]	**<0.05**
Flexibility with “sit-and-reach test”	7.5 (±7.76)	8.31 (±7.45)	[−2.30, 1.24]	0.53
VO_2_ Max (ml/kg/min)	M 29.92 (±9.18)	M 28.21 (±9.89)	[26.37, 30.05]	
	F 30.01 (±3.26)	F 29.55 (±1.84)	[24.77, 34.33]	
	T 29.83 (±8.99)	T 28.54 (±8.53)	[27.07, 30.02]	**0.08**
Biological outcomes (M, SD)				
Lipid profile				
TG (mmol/l)	M 2.41 (±2.72)	M 2.40 (±1.90)	[−0.97, 2.08]	0.45
	W 1.26 (±0.44)	W 1.08 (±0.29)	[−0.004, 0.39]	**<0.05**
	T 2.56 (±3.09)	T 2.10 (±1.84	[−0.60, 1.50]	0.38
Tot-Cs (mmol/l)	M 4.83 (±1.18)	M 5.34 (±1.21)	[−0.41, 0.25]	0.61
	W 4.62 (±0.98)	W 4.78 (±0.72)	[−0.41, 0.15]	0.27
	T 4.91 (±1.19)	T 5.01 (±1.18)	[−0.33, 0.14]	0.41
LDL-Cs (mmol/l)	M 2.65 (±0.89)	M 3.14 (±0.71)	[−0.85, 0.40]	0.45
	W 2.59 (±0.93)	W 2.76 (±0.78)	[−0.63, 0.24]	0.30
	T 2.61 (±0.91)	T 2.82 (±0.67)	[−0.63, 0.21]	0.30
HDL-Cs (mmol/l)	M 1.10 (±0.27)	M 1.05 (±0.26)	[0.008, 0.09]	**<0.05**
	W 1.41 (±0.38)	W 1.40 (±0.31)	[−0.26, 0.21]	0.79
	T 1.17 (±0.27)	T 1.14 (±0.31)	[−0.03, 0.09]	0.34
Carbohydrate balance sheet				
FBS (mmol/l)	5.1 (±1.14)	5.09 (±0.99)	[0.24, 0.31]	0.78
According to the type of psychopathology:				
Anx-MDs	4.82 (±0.44)	5.06 (±0.41)	[−1.14, 0.65]	0.50
Psychosis	4.85 (±0.86)	4.99 (±0.61)	[−0.05, 0.73]	**0.07**
Specific analyses				
Lep (ng/ml) Psychopath.:	13.06 (±8.54)	16.36 (±20.14)	[−27.00, 17.19]	0.61
Anx-MDs	13.21 (±8.41)	15.01 (±8.85)	[−61.59, 84.02]	0.58
Psychosis	14.23 (±8.01)	12.01 (±7.25)	[−58.57, 75.79]	**0.08**
Ghr (pg/ml)	788.00 (±1,101.54)	684.62 (±676.19)	[−686.38, 674.63]	0.98

Bold values: slightly or significant *p*-values.

Data are expressed as mean (M), standard deviation (±SD), percentage (%), and number of patients (*N*).

MetS, Metabolic Syndrome, (at least three of the following five criteria: abdominal circumference, blood pressure, triglycerides, HDL cholesterol, fasting blood sugar); AC, Abdominal Circumference; BP, Blood Pressure; TG, Triglycerides; TOT-Cs, Total Cholesterol; Anx, Anxiety; MDs, Mood Disorders; PSD, Psychotic Spectrum Disorder; SZ, Schizophrenia; HDL-Cs, High-density lipoprotein cholesterol; CGI, Clinical Global Impression; M, men; W, women.

These 24 patients had a mean age of 25.7 years (±7.5; 66.7% men). Among them, 93.8% were single, 56.3% were Swiss or European, 43.8% lived alone, and 79.2% were overweight or obese (respectively, 25% and 54.2%), with a 37.5% prevalence of MetS, 62.5% suffering from anxiety and mood disorders, and 33.3% from psychotic disorders. Most of the subjects were receiving at least one psychotropic treatment (87.5%, and 54.2% were taking an antipsychotic) (Table 2).

#### Differences between men and women

Concerning anthropometric outcomes, univariate tests indicated a significant difference between men and women at baseline and T1. We found no difference in the above variables over time while controlling for gender. No significant differences were observed from baseline to T1 for fat-free mass (kg) [*F* (1.17) = .818, *p* = .66], waist circumference (WC) [*F* (1.19) = .885, *p* = .18], and waist-to-hip ratio [*F* (1.19) = 2.48, *p* = .11].

##### Physical and anthropometric results

Concerning PA, at post-intervention, patients improved their muscular endurance and their coordination (speed of limb movement) from T0 (*M* = 13.65, SD = ±1.93) compared to T1 (*M* = 12.49, SD = ±1.81), [*t*(20) = 3.072 *p* = <0.05]. No significant differences were observed for the other two subscales of the EUROFIT test: flexibility [*t*(20) = −1.026, *p* = .53], nor whole body balance [*t*(20) = .497, *p* = .93] (Table 3).

At the post-intervention point, there was no difference in weight, BMI, or body composition compared to baseline. In particular, we observed no change in fat mass or fat-free mass (lean mass) at T1 compared to baseline [*t*(26) = −.228, *p* = .821]; [*t*(25) = −.646, *p* = .524].

##### Eating behavior results

Concerning the evolution of eating behavior outcomes over time, we observed a slight change in the TFEQ hunger score from baseline (*M* = 7.38, SD = ±3.9) to post-intervention at T1 (*M* = 5.3, SD = ±3.2), [*t*(12) = 2.057, *p* = .06]. No significant difference was observed for the other two subscales of the questionnaire, such as dietary restriction [*t*(15) = −.064, *p* = .95] and disinhibition [*t*(15) = 4.404, *p* = .18] (Table 3).

##### Biological results

Concerning the evolution of biological outcomes over time, we did not observe a significant change in the biological outcomes such as triglycerides [*t*(20) = .−.899, *p* = .38], total cholesterol (Tot-Cs) [*t*(20) = .−.849, *p* = .41], LDL cholesterol (LDL-Cs) [*t*(18) = .−1.074, *p *= .30], HDL cholesterol (HDL-Cs) [*t*(20) = .986, *p *= .34], HDL cholesterol [*F*(1,17) = .818, *p *= .79], fasting blood glucose [*t*(20) = .291, *p* = .78], serum leptin levels [*t*(7) = .−.525, *p *= .61], and serum ghrelin levels [*t*(7) = .−.020, *p *= .98] (Table 3).

##### Psychometric outcomes

Concerning those patients with anxiety or mood disorders, MADRS scores decreased slightly at 8 weeks from baseline T0 (*M* = 1.75, SD = ±.89) to T1 (*M* = 1.30, SD = ±.81), [*t*(2) = 3.272, *p* = .081]. A significant difference was observed for the general well-being (WEMWBS) [*t*(13) = −1.008, *p* = .05] from T0 (*M* = 44.23, SD = ±7.8) to T1 (M = 47.41, SD = ±6.7), with patients with anxiety, depression, and psychotic disorders increasing their general well-being from T0 (*M* = 47.75, SD = ±5.12; *M* = 41.80, SD = ±9.98) to T1 (*M* = 52.00, SD = ±5.10; *M* = 50.60, SD = ±3.78) (*t*(3) = −4.12 *p* = .026; *t*(4) = −2.91 *p *= .044).

##### Differences by diagnosis

There was a significant effect of the program on mental well-being according to the type of psychopathology [*F*(3,10) = 4.25, *p* < .05]. In particular, patients with anxiety-mood disorder symptoms increased their general mental well-being (WEMWBS) from T0 (*M* = 47.75, SD = ±5.12) to T1 (M = 52.00, SD = ±5.010) [*t*(3) = −4.123, *p *= .03]. Concerning patients with psychosis, no significant difference was found for the PANSS total score [*t*(4) = −1.51, *p *= .205] and a tendency for a decrease in abdominal circumference was observed at T1 (*M* = 108.50, SD = ±15.46), [*t*(7) = 2.11, *p *= .072] compared to T0 (*M* = 110.75, SD = ±14.73). No difference in abdominal circumference (AC) was observed at T1 in patients with mood disorders [*t*(7) = −1.340, *p *= .222] or in those with anxiety disorders [*t*(4) = 1.00, *p *= .374].

A tendency towards a decrease in plasma leptin levels in patients with psychosis from the baseline (*M* = 9.56, SD = ±9.68), to the end of the program (*M* = 12.11, SD = ±10.51), [*t*(2) = −3.32, *p *= .080], was observed in addition to a slight (not significant) change in glucose from T0 (*M* = 5.24, SD = ±.400) to T1 (*M* = 4.90, SD = ±.707), [*t*(6) = 2.16, *p* = .074]. No significant difference in plasma leptin levels or blood glucose levels was observed in patients with anxiety-depression symptoms [*t*(7) = 2.11, *p *= .072] (Table 3).

Finally, no difference over time was observed in CGI_Gravity or CGI_Improvement from baseline T0 (*M* = 4.07, SD = ±1.07) compared to T1 (*M* = 4.00, SD = 1.36) and to T2, after 4 weeks after the end of the program (*M* = 2.92, SD = ±1.75), *p *= 0.86 and *p *= 0.36, respectively (Table 4).

**Table 4:** Psychometric outcomes over time: baseline, T1, and T2 (after 4 weeks after the end of the program). Data are expressed as mean (M), standard deviation (±SD).

	Pre-intervention	Post-intervention T1	Follow-up T2	95% CI	*P*-value
CGI_Gravity (M, SD)	M: 4.25 (±1.06)	M: 3.92 (±1.50)	M: 3.11 (±1.76)	[3.34, 5.17]	0.86
	F: 4.20 (±1.095)	F: 2.67 (±1.37)	F: 2.5 (±1.91)	[−2.19, 8.19]	
	Total: 4.07 (±1.07)	Total: 4.00 (±1.36)	Total: 2.92 (±1.75)	[−2.08, 2.99]	
CGI_Improvement (M, SD)	M: 2.83 (±1.27)	M: 3.08 (±0.86)	M: 2.92 (±1.75)	[2.63, 3.74]	0.36
	F: 3.00 (±2.2)	F: 2.67 (±1.36)	F: 3.54 (±1.39)	[2.00, 2.00]	
	Total: 2.64 (±1.28)	Total: 2.93 (±0.99)	Total: 3.54 (±1.39)	[2.75, 3.01]	

CGI, Clinical Global Impression.

## Discussion

A pilot study was conducted to explore the feasibility of the 4-F program as a real-world intervention. A total of 24 outpatients suffering from moderate to severe psychiatric disorders completed the 8-week intervention. Despite the limited sample size, a significant improvement in muscular endurance and coordination and an increase in general mental well-being were observed in these patients from baseline to T1, while depression scores slightly decreased in the 8-week program. Meanwhile, no significant decreases in weight, BMI, or body composition were observed. Only the subgroup of patients suffering from psychosis showed a slight decrease in the abdominal circumference (AC). A slight change in eating behavior was observed over time, with a tendency towards a decrease in the TFEQ hunger scores.

Patients who completed the 8-week program improved muscular endurance and coordination, with no change in flexibility or whole-body balance. In line with previous reports (43), the Eurofit test battery was used to evaluate physical fitness parameters in patients with schizophrenia or schizoaffective disorder. Both groups of patients had impaired speed of limb movement, strength, and abdominal muscular endurance compared to the healthy controls. This demonstrates that patients with psychiatric diseases have poor physical performance, exacerbated by unhealthy lifestyle habits, diet, and sedentary behavior; in addition, increasing illness duration may be a strong correlate of performance on several Eurofit test items. Possible reasons for the association of poorer physical fitness with longer illness duration include the cumulative long-term effect of poor health behaviors such as physical inactivity in patients who often suffer from psychomotor slowing. The Eurofit test may also be a valuable tool for evaluating physical fitness in inpatients with bipolar disorder, as some authors have reported significant correlations between Eurofit test items and age, illness duration, body mass index, smoking behavior, mean daily lithium dosage, and depressive and lifetime hypomanic symptoms (44).

In the 4-F program, a deliberate choice was made regarding the dosage of exercise. An 8-week group-based intervention was implemented following the recommendations of the World Health Organization (WHO) and the American College of Sports Medicine (ACSM) (45). The program consisted of two sessions per week, each lasting 60 min. One session included progressive, moderate-intensity aerobic exercise, while the other included ball team sports. It is worth noting that in the recent literature, various exercise groups have been studied, ranging in duration from 2 to 52 weeks (29, 46), with an average of 12 weeks (47). Session duration ranged from 10 to 90 min, with an average of 60 min, and frequency ranged from one to four times per week (48, 49). The quality and intensity of the physical activity varied, and it included low-intensity exercise (such as yoga or similar), moderate-intensity exercise (50), and moderate-to-vigorous intensity exercise (aerobic training) (51).

Despite some discrepancies in methodological design, these interventions had a real impact on the physical and mental health of patients suffering from severe psychiatric disorders.

It appears crucial to address their motivation for non-adherence with behavioral and motivational counseling (52).

An improvement in general mental well-being from baseline to post-intervention and a slight positive change in depression scores were observed in the 8-week program, while no difference in positive or negative scores on the PANSS was noted. This is in line with a previous 12-week program consisting of moderate-to-intense aerobic PA in which the authors observed enhanced cardiorespiratory fitness, well-being, and reduced psychiatric symptoms (53). In a longer 12-week randomized controlled study with PA interventions on first-episode psychotic patients, the authors reported a decrease in positive symptoms of PANSS and overall psychopathology. Additionally, exercise was found to be protective against the increase in negative scores observed in the control group (54).

This is consistent with a randomized control study that reported better weight management in the intervention group (behavioral interventions, diet, and exercise) in drug-naïve patients with first-episode psychosis in the first three months following the introduction of atypical antipsychotic drugs compared to a control group (55).

Not surprisingly, in the final sample of this study, 79.2% of the subjects were overweight or obese, and the prevalence of MetS was 37.5%. In the general population, this constellation of risk factors has been associated with the development of cardiovascular disease (56). This result is consistent with the results of a previous meta-analysis (57), which found that almost one in three unselected patients with schizophrenia met the criteria for MetS, and a study of bipolar and unipolar depressed patients who had a higher prevalence of MetS and BMI (BD = 46.9% vs. MDD = 35.1%, vs. general population = 22.1%) compared to the population sample (58). A recent meta-analysis showed that the prevalence of MetS in 18-year-old subjects with depression was 31.8% (59). This was in contrast to previous work conducted in 2009, wherein in a cohort of 153 psychotic patients, 46.4% were overweight or obese, while only 19% of them had MetS (19). These results indicate that before encouraging individuals with psychiatric disorders and who are overweight to increase their PA levels, it is essential to quantify their physical health and low PA levels. It is also important to establish a realistic and gradually attainable objective with them in terms of frequency, intensity, and duration of the exercises (60). The American College of Sports Medicine (ACSM) has defined the recommended exercise dosage, taking into account the frequency, intensity, time, and type of exercise as determinants of dosage (45).

Interestingly, for this cohort, no significant decrease in weight, BMI, WC, or body composition was observed. This may be due to the short duration of the program, the low participation rate, and the characteristics of the population studied (most of them were overweight or obese with multiple diagnoses).

In contrast, in an 18-month controlled study, the active group with a weight control program that included an educational activity significantly reduced their body weight and WC (61). In fact playing a team sport requires anticipation and speed to understand the actions of team members (22). This is consistent with previous reports from a 12-week randomized controlled study on psychotic patients, where the authors found no change in BMI after the PA intervention (53). In addition, a previous report of patients with first-episode psychosis who completed a 14-week aerobic interval training program found improved metabolic outcomes and cardiorespiratory fitness after the intervention, although their clinical status remained moderately to severely symptomatic and functionally impaired (25). Meanwhile, depression scores decreased slightly in the 8-week program. Several clinical studies have investigated PA programs for mood disorders and suggested that exercise can alleviate both depressive and anxiety disorders (62).

However, in this study, only the subgroup of patients suffering from psychosis had a slight decrease in the abdominal circumference (AC) after 8 weeks of the program. These results were confirmed in a 14-week aerobic interval training program with 25 individuals with a first psychotic episode. The authors observed the effectiveness of aerobic training in reducing weight and waist circumference while also improving maximal oxygen uptake (25). In the 14-week AIT study, greater WC reduction and weight loss were reported, whereas in a previous study, it took 12–18 months and 3–6 months, respectively, to obtain the same magnitude of reduction in WC and weight (61).

This suggests that AIT may induce a faster reduction in WC than traditional exercise training in a psychiatric population, as has been demonstrated in the general population (54). However, psychiatric population characteristics, such as lack of motivation or psychomotor slowing, should be considered when implementing a progressive intensity dosage.

No improvement in patients' VO_2_ max was observed at the end of the program, whereas other authors reported a significant increase in VO_2_ max in a 14-week aerobic interval training (AIT) program (25). This result was confirmed in patients with metabolic syndrome, where VO_2_ max increased more after AIT than after moderate continuous training after 16 weeks and was associated with the elimination of more risk factors constituting the metabolic syndrome (54). In a 4-week, randomized, single-blind, controlled clinical trial in 57 patients with depression who received add-on aerobic exercise or no activity, the authors reported that VO_2_ max and O2 pulse parameters increased over time only in the exercise group and remained unchanged in the control group (63).

In this 8-week intervention, there was no significant improvement in laboratory parameters (lipid profile or fasting glucose) over time, as in a previous controlled study of an exercise and dietetic program conducted in young patients with first-episode psychosis (24). Since the onset of the disease represents a critical period to prevent side effects and metabolic morbidities, it is crucial to implement effective lifestyle habits and acquire life skills through multidisciplinary training as routine care and include them in their routine care (as quickly as possible from the onset of the disease) from the initiation of treatment in young patients with serious mental diseases (24). This result must take into account that most of the patients had at least one psychotropic treatment (87.5%, and 54.2% benefited from an antipsychotic one), which differs from prior reports that observed blood lipid or glucose dysregulations when the patient was under atypical AP (64).

Interestingly, a slight decrease in the TFEQ hunger score was observed from baseline to post-intervention at T1, in line with previous works, which reported that the TFEQ disinhibition and hunger scores increased according to body mass index (19). PA may represent a valuable factor and predictor to help change the physiological imbalances caused by several psychiatric disorders and unhealthy lifestyles (65). There is evidence that exercise may influence the drive to eat through the modulation of hunger (66).

Finally, the effects of the 4-F program on clinical global impression (gravity and improvement) did not differ after 4 weeks of intervention, in line with a previous study showing that improvements on the Calgary Depression Scale (CDS) in both yoga and aerobic exercise groups at 3 months of intervention remained stable at 18 months of follow-up in both intervention groups (67).

## Limitations

The results of this pilot study demonstrate the feasibility of the 4-F program in assessing 8 weeks of adapted physical activity levels and improvements in muscular endurance and coordination in young patients with moderate-to-severe psychiatric disorders. Additionally, it highlights a notable increase in the overall mental well-being of these patients.

However, there are some limitations to this study. First, the hypotheses could not be tested due to a lack of adequate power and sample size calculations.

Second, the study was non-randomized and non-controlled due to the absence of an active control group. Nevertheless, the effect of a specific 8-week program on mental and physical outcomes was evaluated in an outpatient setting with a 4-week follow-up.

Third, the longitudinal analyses were conducted on a small sample size of participants; therefore, the results and conclusions should be regarded as preliminary and do not allow for definitive conclusions to be drawn. The dropout rate reached the upper limit of the range reported in previous findings [20 to 50% in outpatients (41)], and there were occasional missing data in some items of the scale, which could be attributed to multiple parameters. These include a lack of motivation and attrition due to the mental disease itself, outpatient inclusion, the COVID-19 outbreak with the subsequent quarantine periods, the low priority given to physical activity by psychiatrists, and other barriers that hindered engagement in the exercise program, such as the organization of daily routines (68). In addition, no significant differences were found when comparing the dropout rates between the three diagnosis groups. Response to physical activity was highly individualized, making it difficult to satisfy everyone. Creative ways, optimized motivation, and daily practice activities to improve mobility, coordination, and strength (to develop motor skills) are needed to monitor and enhance adhesion and compliance in this population (69, 70).

Finally, the Eurofit test as a speed of limb movement by plate tapping is expected to have a large learning effect when performed for a second time, so these preliminary results should be interpreted with caution (40, 43). Despite these limitations, several results indicate that an innovative program is feasible and can lead to an effective improvement in muscular endurance and coordination, increasing well-being over time. Moreover, this pilot study was conducted with a pragmatic and real-world approach, aiming to be more inclusive in terms of diagnosis, initiation of exercise, promotion of physical health, encouragement of healthy lifestyle habits, and prevention of weight gain and metabolic dysfunction.

The feasibility of this program will help us implement and develop a structured research plan, considering a larger cohort of patients with a control group, to strengthen our methodology and obtain strong results.

## Conclusion

Despite the acknowledged limitations, these preliminary findings suggest that the implementation of an 8-week group-based program may be feasible and may lead to potential increases in muscular endurance and coordination, along with improvements in the general mental well-being of patients compared to baseline. This pilot study has the potential to guide the refinement of procedures to foster better patient involvement and adherence to the program, thus potentially reducing dropout rates. It also suggests the benefits of a multidisciplinary approach that focuses on both physical and mental health.

Additionally, the outcomes of this study provide a rationale for initiating subsequent longitudinal follow-up research in a larger and more controlled cohort of patients. Such extended investigations could significantly advance the understanding of these interventions, which have already shown promise in improving the quality of life and reducing morbidity and mortality in individuals dealing with severe mental illnesses.

## Data Availability

The raw data supporting the conclusions of this article will be made available by the authors, without undue reservation.
